# eIF3: a critical player in mRNA recruitment to the ribosome with emerging roles across translation

**DOI:** 10.1042/BST20253069

**Published:** 2025-12-24

**Authors:** Nicholas A. Ide, Sufana Noorwez, Christine Xu, Colin Echeverría Aitken

**Affiliations:** 1 Department of Biological Sciences, Columbia University, New York, NY, U.S.A.; 2 Present Address: Department of Genetics, Harvard Medical School, Boston, Massachusetts, USA; 3 Biochemistry Program, Vassar College, Poughkeepsie, NY, U.S.A.; 4 Biology Department, Vassar College, Poughkeepsie, NY, U.S.A.

**Keywords:** biochemistry, eukaryotic gene expression, molecular mechanisms, MRNA, ribosomes, translation

## Abstract

Eukaryotic translation initiation factor (eIF) 3 is a multi-subunit protein complex that plays critical roles throughout translation initiation and has been implicated in a variety of human diseases. More recently, eIF3 has been tied to translation elongation and termination, as well as translational regulation. And yet, a mechanistic understanding of how eIF3 and its constituent subunits perform their canonical roles during initiation continues to elude us. Work across the last two decades has delineated broad mechanistic roles for some of these subunits and identified distinct modules of the complex that contribute differentially to the recruitment of messenger RNA (mRNA) to the ribosome during initiation. Structural approaches have further illuminated these putative roles. And yet, key mechanistic questions tied to fundamental technical challenges remain. Even so, new developments are poised to address these challenges and push our understanding of eIF3 function forward in the coming years.

## eIF3 is a central player in translation initiation and has been implicated in events throughout translation

**Figure 1:**
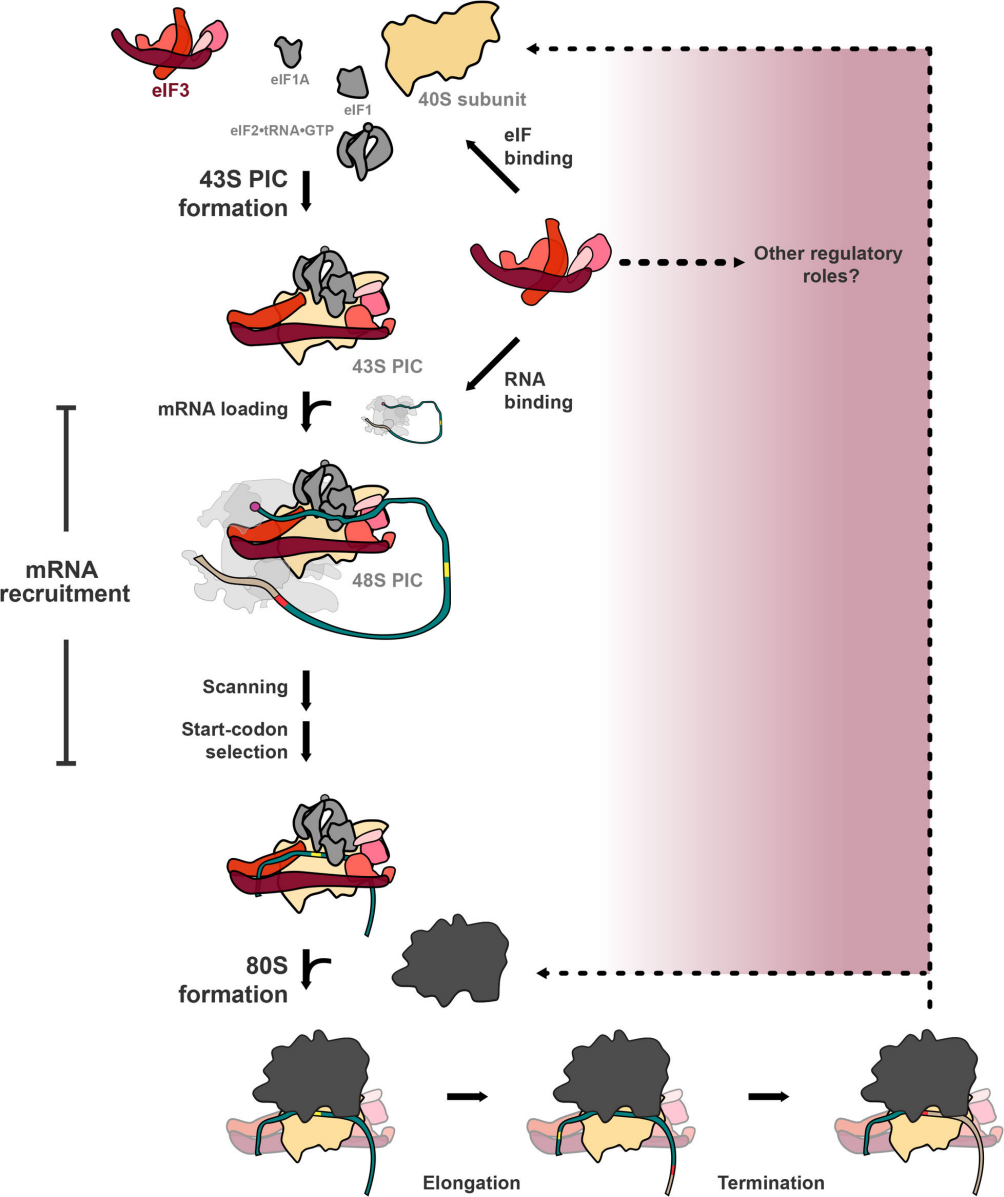
eIF3 is a central player in translation initiation and has been implicated in events throughout translation. eIF3 mediates steps across translation initiation, including formation of the 43S pre-initiation complex (PIC) and the component events of mRNA recruitment. More recent evidence suggests that it contributes to subunit joining, translation elongation, translation termination, and recycling. In addition, eIF3 has been implicated in a number of other regulatory functions, whose relation to its roles in translation initiation remains unclear.

**Figure 2:**
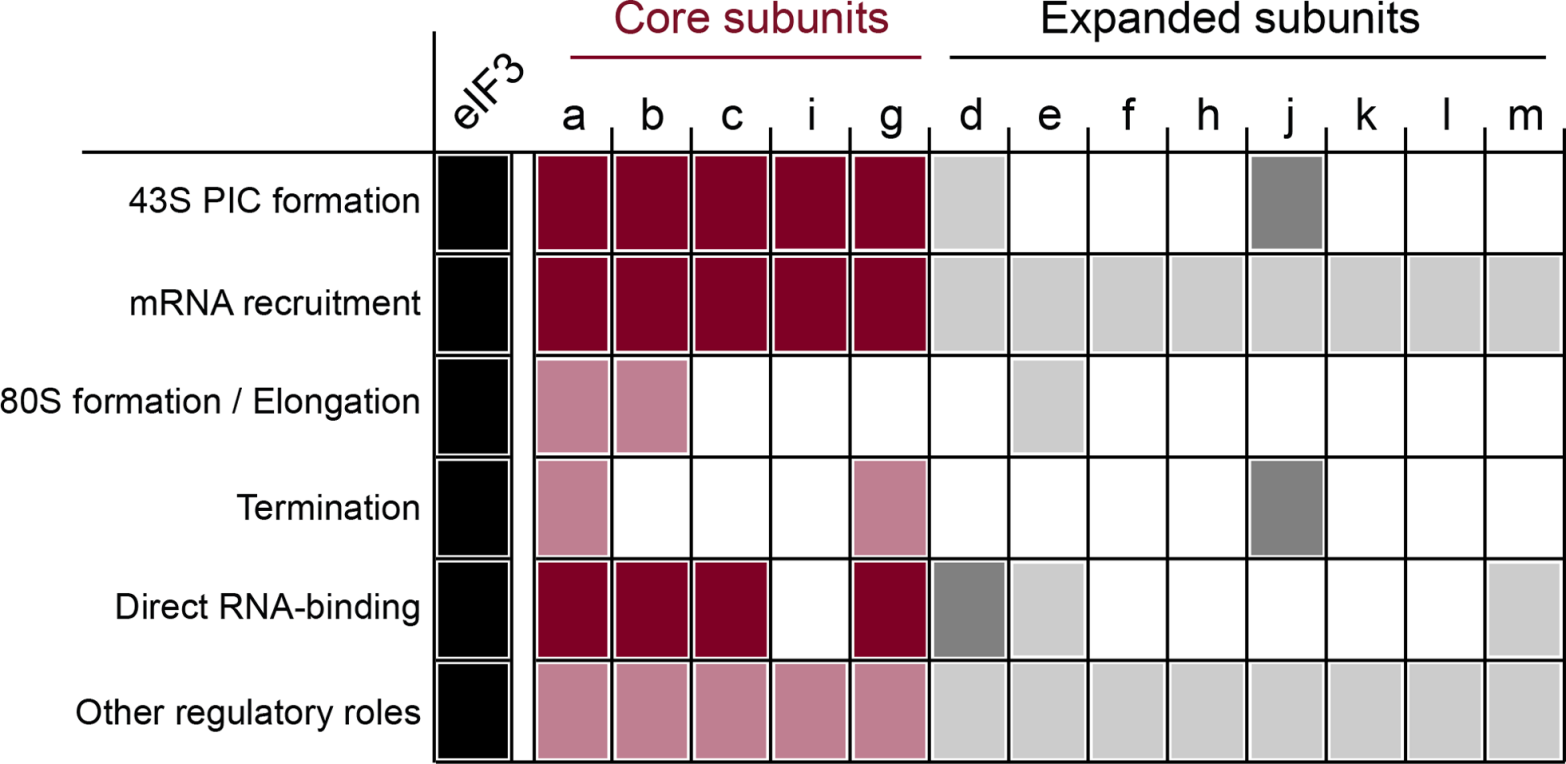
eIF3 has been tied to multiple roles, but the individual contributions of its subunits are less clear. A non-exhaustive table summarizing which subunits have been mechanistically associated with the various roles of eIF3. In nearly all cases, the contribution of the subunit is thought to be in the context of a full eIF3 complex, but this remains largely unexplored. Subunits of the core complex are shown in red, and expanded subunits are in gray. Darker boxes denote that clear evidence of a direct mechanistic role for the individual subunit in the indicated function is present in the literature. Lighter boxes denote correlative or otherwise indirect evidence. Empty boxes denote that we are unaware of published evidence reporting a role.

Translation of messenger RNA (mRNA) into protein by the ribosome is the final core step in gene expression and a key regulatory checkpoint controlling cell fate and response to stimuli [1–4]. Translation initiation is the rate-limiting and most highly regulated step of mRNA translation [5–8]. In eukaryotes, translation initiation is a multi-step process requiring a host of participants: the mRNA, the initiator tRNA, the ribosome, and more than a dozen eukaryotic translation initiation factors (eIFs). The largest and most complex of these factors, eIF3, participates in events throughout initiation and interacts with multiple other initiation components and the ribosome. Depending on the species, eIF3 is composed of 5–12 subunits and ranges in mass from 360 to 800 kilodaltons [9,10]. It contributes to the formation of a ribosomal pre-initiation complex (PIC) [5,11], loading of the mRNA into the PIC [4,12], scanning and selection of the appropriate start codon [13], and joining of the large ribosomal (60S) subunit to form the 80S initiation complex (IC) [14,15] (Figures 1 and 2).

Consistent with these diverse mechanistic contributions, eIF3 interacts with multiple components of the initiation machinery. These observations have led to the suggestion that it might co-ordinate events and interactions during initiation [8,10]. Within the 43S ribosomal pre-initiation complex (43S PIC), eIF3 interacts with the 40S subunit, eIF1, eIF5, and the eIF2•tRNA•GTP ternary complex (TC) [12]. These interactions are thought to stabilize a conformation of the PIC that is competent for mRNA loading [1,11–13,16]. In parallel, the mRNA is activated for loading onto a 43S PIC by the heterotrimeric protein complex eIF4F comprising eIF4A, eIF4E, and eIF4G [7,17]. In mammals, eIF3 interacts with eIF4G [18–22] and thus may bridge the 43S PIC and the activated mRNA. In yeast, this interaction has not been reported [23], but yeast eIF3 may functionally interact with eIF4F via its ability to stimulate the ATPase activity of eIF4A [24].

Loading of the mRNA onto the ribosome forms the 48S PIC, which must then scan the mRNA in the 5′–3′ direction until it identifies the correct start codon [13]. Mutations in multiple eIF3 subunits elicit defects in scanning and/or start-codon selection (Figure 2) [25], suggesting that it also contributes to these steps, perhaps via its interactions with eIFs 1, 2, and 5 [26–28] or via its influence on the conformation of the PIC itself. Together, mRNA loading, scanning, and start-codon selection constitute the overall process of mRNA recruitment (Figure 1), for which eIF3 is required both *in vivo* and *in vitro*. In cells in which the eIF3 complex is degraded, the association between 40S subunits and mRNAs is lost [29]. In a reconstituted *in vitro* system, no detectable mRNA recruitment is observed in the absence of eIF3 [23,25].

Structural approaches have shed light on the physical origins of the roles of eIF3 throughout mRNA recruitment, revealing that it binds the PIC as two distinct modules. One of these modules is present where mRNA enters the ribosome and another where the mRNA exits [10,26–28,30–32]. Biochemical experiments have begun to illuminate the mechanistic contributions of these modules [21,25], but the physical connection between them has yet to be structurally resolved, and their individual contributions have yet to be investigated *in vitro*. Whether, or how, these modules communicate and collaborate remains a mystery.

Once the 48S PIC identifies and pauses at the correct start codon on the mRNA, a series of conformational rearrangements within the PIC clears the way for the large ribosomal subunit to join and form an elongation-competent 80S IC [5,15] (Figure 1). One of the earliest described functions of eIF3 was its anti-subunit joining activity [33,34], presumably serving as a checkpoint between start-codon recognition and 80S formation. Recent evidence suggests that a conformational rearrangement of eIF3 enables subunit joining and might explain how it appears to remain bound to elongating ribosomes in some contexts [26,28,31] (Figure 2).

In fact, the reported activities of eIF3 extend beyond initiation, with reported roles in translation elongation [35–38], termination [35,39–41], and binding of specific mRNA elements, modifications, or other RNA-binding proteins [36,42–45] (Figure 2). Consistent with this, eIF3 has been tied to interactions with numerous molecular players that participate in events across translation and its regulation [9,36,40,42,46–64]. These observations have led to the proposal that eIF3 may serve as an orchestrator of translation, much like the mediator complex co-ordinates transcription [65].

While genetic, biochemical, and structural approaches have recently illuminated key roles played by eIF3, they have also underscored how little we know of the mechanism that underlies them. The mechanistic contributions attributed to the various subunits by genetic and biochemical studies in extract or *in vitro* are varied and overlapping: mutations targeting one subunit do not map onto discrete steps in translation but instead affect multiple steps [9,25,62,66–68] and the effects observed when targeting one subunit often overlap with those observed for mutations in other subunits or even other initiation factors [6,9,12,13,25,68]. In fact, individual eIF3 subunits have been implicated in numerous physical or functional interactions [18,40,45,47,53,56,57,60,62–64,67,69–74] that are assumed to occur within the context of the full complex despite the lack of direct evidence that other eIF3 subunits participate. Delineation of a molecular mechanistic framework for the roles of eIF3 during initiation, as well as throughout the translation cycle and its regulation, will require dissection of the mechanistic contributions of its subunits on and off the ribosome.

Given its fundamental role in the mechanisms of translation, dysregulated activity of eIF3 is unsurprisingly associated with a wide array of human diseases, including a host of cancers [49,75–81], neurodegenerative diseases [82–84], and viral infections [48,74,85–87]. However, a framework tying these putative roles in disease development or progression to specific disruption of the mechanistic contributions of eIF3 is lacking. Such an understanding would likely depend on a clearer description of the mechanistic contributions eIF3 makes to translation initiation and other events during translation.

## eIF3 is compositionally diverse but built around a conserved core of subunits

The expanding range of functions attributed to eIF3 is mirrored by its composition, which is itself expanded across eukaryotes. This composition is built around a conserved core of five essential protein subunits: eIF3a, b, c, i, and g. Together, these constitute eIF3 in the yeast *Saccharomyces cerevisiae*. In many multicellular eukaryotes, this core contains additional subunits, yielding a 12-subunit complex [9,65,82,88–91]. And yet, the subunit composition of eIF3 does not appear to strictly correlate with biological complexity or evolutionary clade. For example, in the yeast *Schizosaccharomyces pombe*, eIF3 contains five additional subunits, resulting in a composition closely resembling that in mammals [10,61]. Additionally, a study characterizing the subunit composition of several protist species identified species containing a wide range of subunits, with some apparently missing subunits of the otherwise conserved core complex [89].

Years of genetic, biochemical, and structural work have outlined a complex network of interactions between the various eIF3 subunits [19,54,67,68,91–95]. Early work identified two stable subcomplexes: one containing eIF3a and eIF3c and the other eIF3b, eIF3g, and eIF3i [68,96,97]. Subsequent work demonstrated that eIF3a can also interact with eIF3b [56,98]; eIF3b in turn holds eIF3i and eIF3g in the complex via its interaction with the former [99]. While their disruption has been tied in some cases to defects in translation initiation [25,67], how these interactions contribute to the functions of the full complex nonetheless remains unclear. Studies knocking down the expression of various eIF3 subunits in a variety of eukaryotic cell types have shown a range of impacts on the stability of eIF3 complexes [56,91,100–102]. Knockdown of eIF3a and eIF3b results in significant destabilization of other eIF3 subunits in human and yeast cells [29,56], while knockdown of eIF3d and eIF3j shows almost no difference in stability of eIF3 complexes, and knockdown of eIF3c results in accumulation of eIF3 complexes containing only the subunits eIF3a, b, i, and g [100].

eIF3 also binds tightly to several initiation factors. eIF1—a key factor in the formation of the 43S PIC and start-codon recognition—was originally misidentified as a subunit of eIF3 because it co-purified with the rabbit reticulocyte complex [103]. eIF5—which binds the eIF3 complex with high (~10 nM) affinity [23]—persistently contaminates otherwise homogenous preparations of eIF3 [28], as do other factors [104,105]. In contrast, the eIF3j subunit is readily washed away during the purification of the *S. cerevisiae* complex and appears to have roles in ribosome biogenesis that may not involve the other subunits [106], leading to some speculation that it may instead be an eIF3-associated factor [10,39,40,69,107,108], though this interpretation is not universal. Further complicating this compositional and interaction landscape is the fact that eIF3 subunits themselves can stably associate with several other initiation factors outside the context of the full complex [59,71,73] and with a more diverse array of molecular players [42,44,49,86], either in the absence of other subunits of the complex or under conditions where the presence of these remaining subunits has not been explored [32,37,42,85,86].

## eIF3 binds the PIC as two modules present near the mRNA-entry and -exit channels of the ribosome

**Figure 3:**
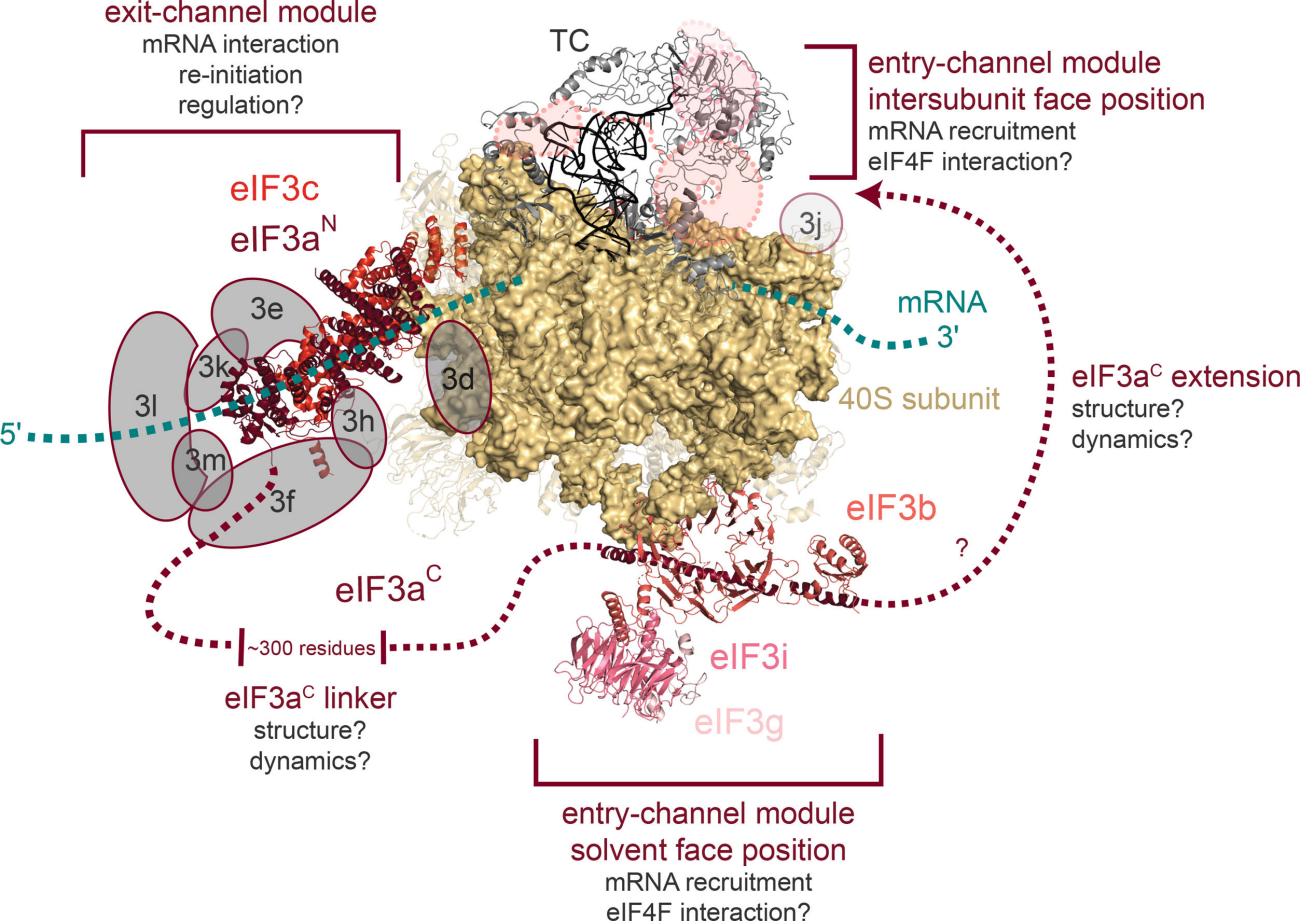
The eIF3 core complex binds the PIC as two modules, one of which is structurally conserved. The structure of a 48S PIC from yeast [31] (PDB ID: 6FYX) is shown with approximate locations of additional human eIF3 subunits within the exit-channel module modeled using a human 48S PIC structure [30] (PDB ID: 6ZMW). To determine approximate locations of the human subunits (cartooned in gray), we aligned the conserved eIF3c subunits from the human and yeast structures. The subunits of the yeast core complex are shown in shades of red, and a second binding location for the mRNA-entry channel module of eIF3 observed in both yeast and mammalian structures (PDB IDs: 5K0Y, 6GSN) is cartooned in salmon (dashed lines near the ternary complex). The ribosomal rRNA is shown as a surface, and all ribosomal proteins are shown as 60% transparent cartoons. Both the mRNA and the unresolved regions of eIF3a are cartooned as dashed lines. Confirmed and putative mechanistic roles, as well as outstanding questions, are indicated next to the corresponding region of eIF3.

Structures of eIF3 bound to the PIC [12,26–28,30,31,59,109–111] have captured eIF3 subunits near action centers of the 43S and 48S PIC, providing clues to its roles in 43S PIC formation and the component events of mRNA recruitment (mRNA loading, scanning, and start-codon recognition). These structures provide a rationale for the essential role(s) eIF3 plays in mRNA recruitment, as they reveal its presence spanning the 40S subunit, placing its subunits near where the mRNA enters and exits the ribosome and where nearly every other initiation factor binds. However, structural studies have yet to resolve the entirety of the PIC-bound eIF3 complex, leaving several key questions unanswered. In particular, large portions of eIF3a, which may serve to communicate between distant regions of the complex, have yet to be resolved (Figure 3). In addition, there are currently no high-resolution structures of the full eIF3 complex free in solution. And yet, a low-resolution structural study comparing the free complex with the complex bound to eIF1 and eIF5 suggests that eIF3 might undergo a large-scale rearrangement upon binding the PIC [54], potentially indicating that conformational dynamics may underlie at least some of the functions of eIF3. Consistent with this, structures of eIF3 bound to the PIC in distinct functional states have been interpreted as suggesting large rearrangements on the ribosome [26–28,31]. How and whether these putative conformational dynamics contribute to the various roles of eIF3 during initiation remains unexplored.

Most structures of eIF3 can be broadly divided into two categories: atomic-resolution structures of eIF3 subunits or individual domains determined using X-ray crystallography [59,63,112–114] or relatively lower resolution structures of the full eIF3 complex bound to the PIC determined by cryo-electron microscopy (cryo-EM) (Figure 3) [26–28,30,31,59,109–111]. The atomic-resolution structures of discrete eIF3 domains or subunits have commonly been fit into the density of PIC-bound eIF3 structures determined using cryo-EM, as the lower resolution of these data has precluded *de novo* building of atomic models from the cryo-EM data themselves.

Together, these structural efforts have defined the locations of eIF3 subunits on the PIC, revealing that eIF3 binds the PIC as two distinct modules (Figure 3). The first of these modules is located near the channel where the 5′ end of the mRNA exits the ribosome and therefore can be termed the ‘eIF3 exit-channel module.’ The core of the exit-channel module is formed by the N-terminal domain (NTD) of eIF3a, which interacts with the eIF3c C-terminal domain [31,59], forming a well-defined heterodimer that constitutes nearly the entire module in *S. cerevisiae* and serves as the foundation for six additional non-core subunits to bind in the expanded complex [26,30] (Figure 3). The NTD of eIF3c extends away from the eIF3a/c heterodimer and inserts itself into the PIC to interact with eIF1 and eIF5, though these interactions have not been well resolved in all structures [27,31,111]. This region of eIF3c also appears to relocate upon start-codon recognition to gate subunit joining [31] and has been suggested to serve as a docking center for the exchange of eIF1 by eIF5 upon start codon selection [71].

The second eIF3 module is found near the channel where the 3′ end of the mRNA enters the ribosome and comprises eIF3 b, i, g, and a portion of the C-terminal half of eIF3a. In contrast to the well-defined structure of the NTD of eIF3a, the structure of the C-terminal half of eIF3a—which mediates interaction with eIF3b to organize this ‘entry-channel module’ and serves as the sole connection between it and the exit-channel module—has eluded structural characterization. It has either been modeled as an extremely long (>300 amino acids) extended ɑ-helical region [30,109–111] (Figure 3) or not at all [26–28,31]. An extended ɑ-helix of this length would measure ~450 angstroms, a distance significantly longer than the 40S subunit itself, highlighting the lack of information regarding the structure and therefore functional contribution(s) of this region of eIF3a. This modeled position and the length of the linker connecting the two eIF3 modules are based on genetic studies [62,67,112] demonstrating that the eIF3a C-terminal half interacts with the eIF3b/i/g subcomplex via eIF3b. In fact, those regions of the eIF3a C-terminal half that interact with eIF3b are not located at the extreme C-terminus of eIF3a, leaving open the possibility that eIF3a projects past the location of the structurally resolved eIF3b/g/i subunits of the entry-channel module (Figure 3). Like the majority of the eIF3a C-terminal half, this very C-terminal extension has yet to be visualized in structural studies. Nonetheless, the C-terminal half appears to be critical to the function of eIF3. Its mutation or truncation is lethal [67], it interacts functionally or physically with elements of the 40S subunit that control the width of the mRNA-entry channel [28,31,59,67] and with the TC [25], and it is the sole point of contact between the two modules bound at distinct ends of the PIC. Moreover, this poorly resolved C-terminal half appears to be largely conserved across eukaryotes. It has similarly escaped visualization in the mammalian complex, and human eIF3a contains a similar and, in fact, longer C-terminal half [30,109,110].

Interestingly, comparison of the two eIF3 modules between yeast and human 48S PIC structures reveals a spatially localized expansion of eIF3 subunits in higher eukaryotes (Figure 3), with a host of additional subunits appearing in the exit-channel module and none in the entry-channel module. This structural conservation of the eIF3 entry-channel module is particularly interesting given that it has been visualized, in both yeast [27,28] and mammalian structures [26], in drastically different positions on the 48S PIC, depending on the functional state of the PIC. Together with the observation that mutations throughout this module affect mRNA attachment, scanning, or start-codon selection (Figure 2), this has led to a model in which positioning (or perhaps movement) of the entry-channel module may play a mechanistic role in these events across eukaryotes [28]. And yet, approaches to directly investigate the dynamic positioning of eIF3 on or off the ribosome have yet to be developed. Such approaches are required if this apparent differential positioning and/or conformational dynamics are to be fully understood.

## Emerging roles for the entry- and exit-channel modules of eIF3

Consistent with the idea that the structural conservation of the entry-channel module might indicate a conserved mechanistic role, yeast eIF3 can stimulate translation initiation in a human cell-extract-based initiation assay [94]. Moreover, mutations affecting start-codon selection fidelity originally identified in yeast have been shown to have similar effects in human cells [100]. Interestingly, despite the structural expansion of the exit-channel module in *S. pombe* and mammals, these additional subunits do not alter the architecture or position of the eIF3a/c heterodimer at the platform of the 40S subunit (Figure 3). However, recent single-molecule experiments suggest that there may be subtle differences in the mechanism of start-codon recognition between humans and yeast, leading to speculation that these might be related to the differential architecture of the eIF3 mRNA-exit-channel modules in these organisms [115]. Nonetheless, the preponderance of evidence points to a model in which the core eIF3 complex forms the functional foundation of the eIF3 complex across eukaryotes and that additional subunits located at the exit-channel module might contribute regulatory roles attributed to eIF3 or perhaps modulate the mechanistic contributions of this module to start-codon recognition or subunit joining.

Consistent with their distinct positions, distinct roles for the entry- and exit-channel modules during mRNA recruitment are beginning to emerge [25]. Truncation of the first 200 amino acids of eIF3a (within the mRNA-exit-channel module) disrupts reinitiation events that depend on specific sequence elements upstream of the AUG codon in yeast [62], and the molecular machinery underlying this interaction appears conserved in mammals [100]. This same truncation also abrogates mRNA binding to the PIC in 48S complexes programmed with an empty mRNA-entry channel (and which thus depend on interactions within the exit channel to stabilize mRNA binding) in a yeast *in vitro* system [25]. Together with structures that show the eIF3a/c heterodimer extending the mRNA-binding surface on the 40S platform [27,28,31,59], this has led to a model in which the exit-channel module plays an important role in stabilizing interactions between the PIC and the mRNA.

In contrast to the exit-channel module, the entry-channel module is not required for stabilizing mRNA binding in the entry channel; 48S complexes programmed with an empty exit channel (and which thus depend on mRNA–PIC interactions in the entry channel) are nonetheless able to remain stably bound to the mRNA in the presence of mutations to the subunits of the entry-channel module [25]. And yet, mutations throughout the entry channel appear to disrupt the processivity of scanning [66,67,93] and significantly slow the kinetics of mRNA recruitment *in vitro* [24,25]. Consistent with this, the entry-channel subunits eIF3i and g have been shown to stimulate the ATPase activity of the eIF4F helicase eIF4A, though the physical interaction underlying this stimulation or its significance to mRNA recruitment remains unknown [24].

In a potentially complementary role, regions of the poorly resolved eIF3a C-terminal half appear to interact with the 40S subunit to modulate the width of the mRNA-entry channel; mutations in this region alter the fidelity of start-codon recognition [67], potentially by modulating the conformational dynamics of the 40S subunit head. However, structural studies have yet to resolve the eIF3a C-terminal half, perhaps because it is intrinsically disordered or dynamic. However, these possibilities—like the functional significance of movement of the entire entry-channel module—have yet to be explored owing to the absence of available assays that directly probe eIF3 compositional or structural dynamics. Similarly unexplored *in vitro* are lethal mutations or truncations (for example, of the eIF3a C-terminal half) identified by genetic studies, owing to the until-recent [47] lack of a reconstituted eIF3 yeast complex that recapitulates the various characterized *in vitro* functions of eIF3.

The apparent functional interaction between subunits of the entry-channel module and eIF4F in yeast (and whether this underlies the requirement for eIF3 in mRNA recruitment) remains mechanistically unexplained, given the lack of evidence for a physical interaction between yeast eIF3 and eIF4F [23]. It further stands in contrast to the observations that human subunits within the opposite module near the exit channel (eIF3e, eIF3c, and eIF3d) interact directly with regions of eIF4G [18,116]. These observations have led to the proposal that the eIF3–eIF4G connection might serve to bridge the PIC and the cap-bound eIF4F complex and thus load the mRNA onto the PIC in human cells [116]. In this model, human and yeast eIF3 would employ distinct networks of interactions to enable PIC attachment during mRNA recruitment. This would be consistent with the proposal that eIF4F remains simultaneously tethered to the 5′ cap and the PIC after mRNA loading in human cells [117], a network of interactions that does not appear to occur in yeast cells [37]. However, this would not explain how yeast eIF3 mediates initiation in a human *in vitro* system [94]. Moreover, a unique and central role for the exit-channel module in a mechanistic event required for initiation on all mRNAs would be surprising, given that its compositional and structural expansion in higher eukaryotes stands in stark contrast to the conservation of the entry-channel module across eukaryotes, which has also been tied mechanistically to eIF4F and mRNA recruitment.

Another possibility is that the entry-channel module might stimulate the recently reported ‘strippase’ activity of eIF4A, which rapidly removes eIF4F bound at cap distal sites of an mRNA but more slowly when eIF4F is bound at the 5′ cap [118]. This location-specific removal of eIF4F appears to facilitate rapid searching of the mRNA to locate and recognize the 5′ cap and could create a kinetic window for the PIC to recognize and functionally interact with the cap-bound complex. A model in which the eIF3 entry-channel module dynamically stimulates eIF4F eviction within the complex of the PIC might explain its previously observed stimulation of eIF4A [24], the lack of an observed stable interaction between eIF4F and eIF3 in yeast [23], and potentially the distinct binding locations observed for the entry-channel module in PICs lacking mRNA or bound to mRNA in pre- or post-start-codon recognition states [28]. This would also explain (i) why yeast eIF3 is required for mRNA recruitment despite not stably interacting with eIF4G as in the mammalian system and (ii) the ability of eIF3 to rescue initiation in a human *in vitro* system [94]. Such a role for the entry-channel module would not preclude subsequent interaction of the exit-channel module with either a second eIF4F or with the original cap-tethered eIF4F after the mRNA has been loaded into the ribosome. Displacement and relocation of the original cap-tethered eIF4F complex would be consistent with the observation that individual eIF4F complexes can be removed from the cap but remain bound to the mRNA, thereby accelerating secondary recognition of the cap [118]. Such displacement and relocation could be facilitated by collaboration between the entry- and exit-channel eIF3 modules, perhaps mediated by the as-yet unresolved eIF3a C-terminal linker that connects them. Regardless of the mechanism of eIF3–eIF4F communication, whether this linker enables communication between these modules (and if so, how) remains an open question. Addressing these questions will depend on the development of *in vitro* techniques that enable physical dissection of these two modules and the subunits they comprise. It will likely also require tools capable of monitoring dynamic structural and/or compositional rearrangements in real time.

## The expanding roles of eIF3 and its subunits beyond canonical initiation

In recent years, the putative functions of eIF3 have expanded well beyond translation initiation Figure 2. Perhaps the earliest described and best studied example of this is the role that the ‘eIF3-associated’ subunit eIF3j plays in translation termination and recycling [10,39,40,106], with several studies reporting across species that eIF3j functionally interacts with the canonical translation termination factors eRF1 and eRF3 [40,69], as well as the ribosome recycling factor Rli1/ABCE1 [69,119]. In fact, the eIF3 core complex has also been implicated in termination and/or stop-codon readthrough (Figure 2), a role that appears to involve components of the 40S subunit with which eIF3 collaborates during mRNA recruitment [41,120].

More recently, a number of studies have tied eIF3 to translation elongation in yeast [10,35,37] and mammals [14,36,49,57], echoing the decades-old observation in plants that eIF3 interacts with either the large (60S) subunit or 80S ribosomes [121,122]. In at least one case, the interaction between eIF3 and the elongating ribosome appears to play an important role in driving the translation of mRNAs important for mitochondrial function [36]. Consistent with this, depletion of eIF3 in yeast appears to preferentially compromise the translation of mRNAs involved in mitochondrial processes [50].

A number of other studies have also detected eIF3 directly interacting with mRNAs [21,23,49,55,60,70,95,114], potentially via post-transcriptional mRNA modifications [43,45,58,123]. In several cases, these interactions have been proposed as potentially driving specific recruitment of these mRNAs to the PIC [45,51,52,57,72,124]. In human cells, for example, several studies have shown that the eIF3d [46,53,72] and eIF3l [21] subunits directly bind the 5′ cap of certain mRNAs and may stimulate non-canonical, eIF4F-independent initiation. Given the participation of eIF3 in translational phases beyond initiation, it is also possible that it may mediate preferential translation of specific mRNAs at a number of steps in translation.

Consistent with this, eIF3 appears to interact with a number of mRNA-binding proteins that participate in events throughout translation [44,61,125–127]. In fact, proteomic investigation has tied these interactions to the compositional complexity of eIF3. Distinct subcomplexes of the eIF3 complex appear to associate with distinct pools of regulatory proteins and mRNAs [57,61,92,128]. This raises the possibility that the composition of the eIF3 complex could be dynamically tuned to modulate the translational landscape. Perhaps consistent with this, the eIF3-associated eIF3j subunit appears to play roles within and without the complex [39,40,69,106] and the cap-binding protein eIF3d, while stably purifying with the complex, makes scant contacts with it and is modeled either outside of the exit-channel module in some structures [30,109] (Figure 3) or is not modeled at all in others in which it was presumably present [26]. Consistent with the idea that certain eIF3 subunits might act outside the context of the eIF3 complex, numerous studies have identified eIF3 subunits that cross-link to or co-purify with other proteins in a variety of pathways outside of initiation [36,52,58,61,124]. In many of these cases, the presence of other subunits has not been directly assessed, or other eIF3 subunits fail to cross-link or co-purify with these species as one would expect if these roles are performed by the entire complex.

## A consensus model for eIF3 function will require subunit-level molecular insight

As the roles attributed to eIF3 continue to expand within translation and beyond, the need to understand the precise mechanistic contributions of its constituent subunits and how these are co-ordinated within the complex becomes increasingly pressing. While it is tempting to speculate that eIF3 may function as a central co-ordinator in translation and its regulation, connecting its many emerging roles to its canonical roles on the ribosome will require a mechanistic framework explaining the roles and interactions of its subunits, both within the core yeast complex and the expanded mammalian complex.

At the very heart of this challenge lies the enduring mystery of how eIF3 drives mRNA recruitment during initiation. To achieve this, eIF3 must bind the 40S ribosomal subunit and interact directly or indirectly with multiple other components of the translational machinery, a list that includes initiation factors, tRNA, and mRNA. In recent years, many of these interactions have been illuminated structurally and interrogated biochemically, but much work remains.

The picture that emerges from this work is a complex that engages the PIC as two primary modules present in the channels where mRNA enters and exits the ribosome. One of these—at the mRNA-entry channel—is structurally and compositionally conserved throughout eukaryotes, stimulates eIF4F (but has not been shown to interact directly with it across species), and is broadly required for mRNA recruitment. The other—at the mRNA-exit channel—is important for stabilizing interactions between the PIC and the mRNA in yeast, interacts directly with eIF4F in mammals [18,116] but not in yeast, and is decorated in higher eukaryotes with subunits capable of cap-binding [46,48,53,72], direct-mRNA binding [42,47,55], or interactions with regulatory proteins [14,44]. However, the evidence connecting each of these modules to the mechanism of mRNA recruitment is largely indirect or circumstantial.

The highly conserved eIF3a subunit is central to both modules across eukaryotes. At the exit channel, it is required for 40S binding [25,129] and stabilizing mRNA–PIC interactions [25]. At the entry channel, its unstructured and unresolved C-terminal half mediates critical interactions with subunits eIF3b, i, and g [67]. eIF3a also serves as the sole connection between the two eIF3 modules (Figure 3). And yet, little is understood about the importance of this connection, and it remains structurally unresolved. How it might enable communication between the modules, with other eIF3 subunits, or with other initiation factors remains a complete mystery. Moreover, how potential movement of eIF3a—particularly of its C-terminal half—might contribute to distinct mechanistic steps during initiation is equally unresolved. And yet, the role of eIF3a and its C-terminal half is one of many questions that remain unanswered. How (and whether) the eIF3b, i, and g subunits of the mRNA-entry-channel module communicate with the eIF4F complex and thereby contribute to mRNA loading or scanning remains an open question. Likewise, the mechanistic origins of the sequence-specific reinitiation events mediated by the eIF3a NTD and eIF3c [35,62] and whether the mechanism of eIF3–eIF4F collaboration during mRNA recruitment is species-specific or conserved remain unresolved.

Establishment of a mechanistic framework for the roles of eIF3 in translation initiation and beyond likely depends on the elucidation of the contributions of these core subunits. This, in turn, will depend on tools for dissecting the roles of the individual subunits and monitoring their dynamics on and off the ribosome. A pervasive barrier to this dissection has been the inability to study lethal mutations or subcomplexes using the various *in vitro* assays available for monitoring sub-steps of translation initiation or molecular interactions important to the pathway. All five subunits of the core complex are essential, and this complex has traditionally been purified natively from yeast for *in vitro* study [23,25]. Thus, individual subunits, subcomplexes, and previously identified lethal mutations have yet to be characterized *in vitro*.

Recombinantly reconstituted yeast [27,54,73] and human [90] complexes have been reported by several groups, primarily for structural studies. A yeast recombinant complex was shown to stimulate translation in an eIF3-depleted cell extract [73] and a human recombinant complex was shown to bind directly to both the hepatitis C virus internal ribosome entry site RNA and to the 40S subunit [90]. Neither of these studies, however, demonstrated that these reconstituted complexes could recapitulate the *in vitro* activities observed for eIF3 within the yeast or mammalian *in vitro* translation initiation systems: 43S PIC binding [25,90,116], stabilization of TC within the 43S PIC [25], mRNA recruitment [23,25], or stimulation of ATPase activity by the eIF4F complex [24].

The recently reported reconstitution of a recombinant eIF3 complex that recapitulates mRNA recruitment and the molecular interactions and activities on which it depends *in vitro* [47] represents a promising step towards resolving at least one of the barriers to a more complete understanding of eIF3. This system should enable independent investigation—using the array of *in vitro* assays that report on key initiation steps—of the mRNA-exit and -entry channel modules and the subunits and domains of which they are composed. It should also illuminate the mechanistic origins of previously identified lethal mutations. Moreover, the ability to completely dissect the eIF3 complex should also enable the preparation of specific subcomplexes for high-resolution structural approaches. Finally, the development of novel single-molecule assays would allow for direct observation of the structural and compositional dynamics of eIF3 that have thus far eluded researchers. Interrogation of eIF3 using proven single-molecule *in vitro* approaches [115,118,130–132], more recently designed *in vitro* tools [47,133], and even *in vivo* techniques capable of detecting the binding and stoichiometry of protein–RNA interactions [134,135] would address a gap in our current understanding. Prime targets for these approaches would be the heretofore unresolved C-terminal regions of eIF3a, the putative conformational rearrangements of the entry-channel module, collaboration between eIF3 and eIF4F, and the role that subcomplexes may or may not play in eIF3 function.

**Perspectives**

- **Importance of Field:** Eukaryotic translation initiation factor (eIF) 3 is a multi-subunit protein complex that is required for recruitment of messenger RNA (mRNA) to the ribosome, the defining mechanistic step of translation initiation in eukaryotes. Dysregulated activity or expression of eIF3 or its subunits is associated with a wide array of human diseases, including a host of cancers, neurodegenerative diseases, and viral infections, but how these outcomes are tied to disruption of eIF3 remains poorly understood.
- **Summary of the current thinking:** eIF3 binds the ribosome as two modules that span the small ribosomal subunit: one structurally and compositionally conserved module present at the channel where mRNA enters the ribosome and another module—expanded with additional subunits in higher eukaryotes—located near the channel where mRNA exits. How these modules and the subunits they comprise contribute to mRNA recruitment and whether (and if so, how) these two modules communicate and collaborate remains mysterious.
- **Comment on future directions:** This central question remains enigmatic due to (i) the inability of structural approaches to resolve key components of the complex, (ii) the lack of *in vitro* tools to fully dissect the mechanistic contributions of the subunits and modules of eIF3, and (iii) a need for assays that directly interrogate potentially dynamic events during mRNA recruitment. The use of biochemically active, reconstituted eIF3 complexes capable of describing the molecular contributions of individual subunits, together with ensemble and single-molecule biophysical approaches, promises to address these limitations and thus shed light on eIF3, its biological roles, and its connection to human disease.

## Open Access

This article has been published open access under our Subscribe to Open programme, made possible through the support of our subscribing institutions, learn more here: https://portlandpress.com/pages/open_access_options_and_prices#conditional

## Abbreviations

IC: initiation complex
PIC: pre-initiation complex
43S PIC: 43S ribosomal pre-initiation complex
TC: ternary complex
cryo-EM: cryo-electron microscopy
eIF: eukaryotic translation initiation factor
mRNA: messenger RNA
